# Adaptive evolution of Hox-gene homeodomains after cluster duplications

**DOI:** 10.1186/1471-2148-6-86

**Published:** 2006-11-01

**Authors:** Vincent J Lynch, Jutta J Roth, Günter P Wagner

**Affiliations:** 1 Department of Ecology and Evolutionary Biology, Yale University, 165 Prospect Street, New Haven, Connecticut 06551, USA; 2 Department of Genetics and General Biology, University of Salzburg, Hellbrunnerstrasse 34, 5020 Salzburg, Austria; 3 National Institute for Medical Research, Division of Developmental Biology, The Ridgeway, London, NW7 1AA, UK

## Abstract

**Background:**

Hox genes code for homeodomain-containing transcription factors that function in cell fate determination and embryonic development. Hox genes are arranged in clusters with up to 14 genes. This archetypical chordate cluster has duplicated several times in vertebrates, once at the origin of vertebrates and once at the origin of gnathostoms, an additional duplication event is associated with the origin of teleosts and the agnanths, suggesting that duplicated Hox cluster genes are involved in the genetic mechanisms behind the diversification of vertebrate body plans, and the origin of morphological novelties. Preservation of duplicate genes is promoted by functional divergence of paralogs, either by subfunction partitioning among paralogs or the acquisition of a novel function by one paralog. But for Hox genes the mechanisms of paralog divergence is unknown, leaving open the role of Hox gene duplication in morphological evolution.

**Results:**

Here, we use several complementary methods, including branch-specific *d*_*N*_/*d*_*S *_ratio tests, branch-site *d*_*N*_/*d*_*S *_ratio tests, clade level amino acid conservation/variation patterns, and relative rate ratio tests, to show that the homeodomain of Hox genes was under positive Darwinian selection after cluster duplications.

**Conclusion:**

Our results suggest that positive selection acted on the homeodomain immediately after Hox clusters duplications. The location of sites under positive selection in the homeodomain suggests that they are involved in protein-protein interactions. These results further suggest that adaptive evolution actively contributed to Hox-gene homeodomain functions.

## Background

The homeobox codes for a highly conserved 60 amino acid DNA-binding motif (the homeodomain) found in transcription factors [1]. One class of homeobox-containing transcription factor genes are the Hox genes, which are homologous to the genes in the Drosophila homeotic (HOM) gene cluster, that specify cell fate during embryonic development [1] and have derived functions in other tissues [2]. Multiple Hox genes located in tightly linked clusters have been identified in all animal phyla examined, with the archetypical chordate cluster having 14 genes (Hox1–Hox14) [3]. The number of Hox clusters has increased several times in vertebrate evolution: the cluster duplicated twice in early vertebrates leading to four clusters (HoxA-D) with 42 genes [4,5] and additional cluster duplications in teleost fish led to 7–8 clusters with 45–47 genes [6,7]. Independent duplications have also occurred in the jawless vertebrates hagfish [8] and lamprey [9].

Models of duplicate gene preservation predict functional differentiation of paralogs based on protein sequence or regulatory divergence [10,11]. Although numerous models of duplicate gene divergence have been proposed, four different mechanisms of functional divergence are likely to explain preservation of duplicate Hox genes: acquisition of novel functions by one paralog (neo-functionalization) [12], passive erosion of functional redundancy due to complementary degenerative mutations, (sub-functionalization) [11], models that predict the accumulation of neutral mutations, which later acquire functional constraints because the environment or genetic background changes (the Dykhuizen-Hartl effect) [13] or divergent adaptive selection of both paralogs (adaptive diversification) [14]. This list has recently been expanded by the introduction of the subneofunctionalization [15] and the adaptive radiation [16] models that predict rapid subfunctionalization after duplication followed by a prolonged period of neofunctionalization and adaptive divergence of duplicate genes in a process analogous to species radiations, respectively. Here, we are interested in testing whether positive selection acted immediately after cluster duplications to promote functional divergence and identify which mechanisms discussed above most adequately explain the preservation of Hox duplicates in vertebrates.

How paralogus Hox genes have been retained is not known, although evidence suggestive of positive selection after cluster duplication has been identified in Hox7 [17], Hox5 and Hox6 [18] paralogs. In these studies, however, it is not clear whether directional selection was responsible for the maintenance of the duplicated genes or other mechanisms promoted the maintenance of duplicates [19]. In addition, evidence for positive selection immediately after Hox cluster duplications has recently been identified in teleost fish for *HoxA-11 *and *HoxB-5 * [20]. These data suggest that, in the evolution of ray-finned fishes, some duplicate Hox genes have been preserved by functional differentiation through the action of positive Darwinian selection immediately following the gene duplication. This suggests that Hox genes may have also experienced adaptive evolution following the cluster duplications earlier in vertebrate evolution.

Hox cluster duplication and gene diversification has been proposed to be one of the genetic mechanisms behind the diversification of vertebrates and body plans and the origin of morphological novelties [21-23]. This association, however, is difficult to reconcile with the perceived degree of sequence conservation between the homeodomains of Hox genes and the numerous examples of functional equivalence of Hox/Hom genes from strikingly divergent organisms [24-28]. Mouse HoxA-5, for example, is able to activate the same target genes as its *Drosophila *homolog, Sex combs reduced (Src), in axis determination indicating strong conservation of function over 500 to 600 million years [29], but counter examples also exist, showing functional non-equivalence of Ubx orthologs from fairy shrimp, velvet worm and Drosophila [30] and non-equivalence of homeodomains from HoxA-4, HoxA-10, HoxA-11 and HoxA-13 paralogs from mouse [31,32].

In this paper we investigate the sequence divergence in homeoboxes from the four gnathostome Hox clusters, including genes from basal vertebrates and sarcopterygians like shark and coelacanth, respectively. This is the first study of homeodomain divergence with extensive taxon sampling allowing us to identify the relative phylogenetic age of substitution events in vertebrate phylogeny. We use three different, but complementary, approaches to test for functional divergence among paralogs: comparison of patterns of amino acid sequence conservation/variation among paralog clades, *d*_*N*_/*d*_*S *_ratio tests to detect directional selection and identify positive sites, and comparison of clade level polymorphisms/divergence rates. Our results indicate that after cluster duplication positive Darwinian selection acted on the homeodomain of Hox proteins prior to the divergence of the modern gnathostome and bony fish lineages. We find amino acid substitutions at sites that are not involved in structural constraints and are located on the molecular surface where they are available for protein-protein interactions were targets of positive selection. We suggest that the action of positive selection at a subset of sites not constrained by ancestral (plesiomorphic) functions after cluster duplications led to the emergence of novel protein interactions while maintaining ancestral ones. This model can help reconcile the role of Hox genes in morphological diversification and innovation with their extreme sequence conservation.

## Results and discussion

### Functional divergence of paralog-group homeodomains

**Figure 1:**
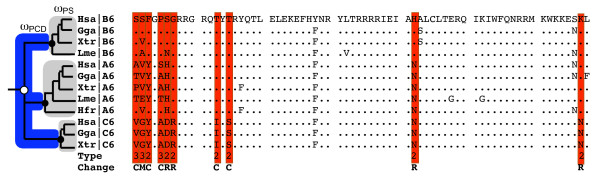
An Example of Functional Divergence in Hox6 Paralogs. The phylogeny of the genes is shown on the left with the location of the cluster duplication indicated with an open circle and speciation events indicated with a closed circle. Post cluster-duplication branches (PCD) and post speciation branches (PS) are highlighted blue and gray, respectively. These branch types were used in the calculation of ω_PCD _and ω_PS _in two ratio analyses for all paralog groups. The amino acid sequence of Hox6 genes from human (Hsa), chicken (Gga), frog (Xtr), coelacanth (Lme) and shark (Hfr) are shown on the left with divergent sites highlighted in red. Below the alignment sites are identified as type-I (1), type-II (2) or both (3). Amino acid substitutions are classified as conservative (C), moderate (M) or radical (R).

**Figure 2:**
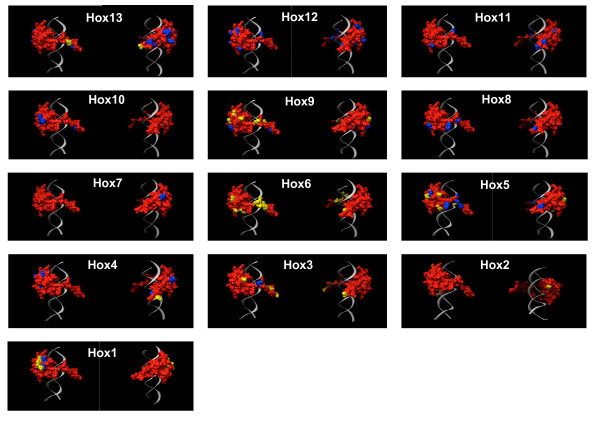
Location of Cluster-Specific Amino Acids on the Molecular Surface of Hox Homeodomains. The homeodomain is shown with the molecular surface in red and DNA in gray. Cluster-specific amino acids are shown in blue and amino acids that were under positive selection after cluster duplications are shown in yellow. Only those sites with a posterior probability larger than 0.90 of having ω > 1 are shown in yellow.

We compiled a database of Hox genes with 4–5 species for each gene (155 sequences in total) and compared conserved and variable sites between paralog group members to identify if there are characteristic residues that distinguish which cluster a paralog belongs to (for example, see Figure 1). This analysis identified many sites that are conserved among species but variable between genes in the same paralog group ('cluster-specific' residues; Figure 2). Although the homeodomain is a highly conserved motif, it is not invariant; in fact only 17 residues are absolutely conserved between all vertebrate Hox genes in our alignment, suggesting that variable sites could be functionally divergent. Many of these variable sites have been previously shown to be 'characteristic residues' that distinguish paralog groups from each other and have been suggested to be engaged in protein-protein interactions [33].

**Figure 3:**
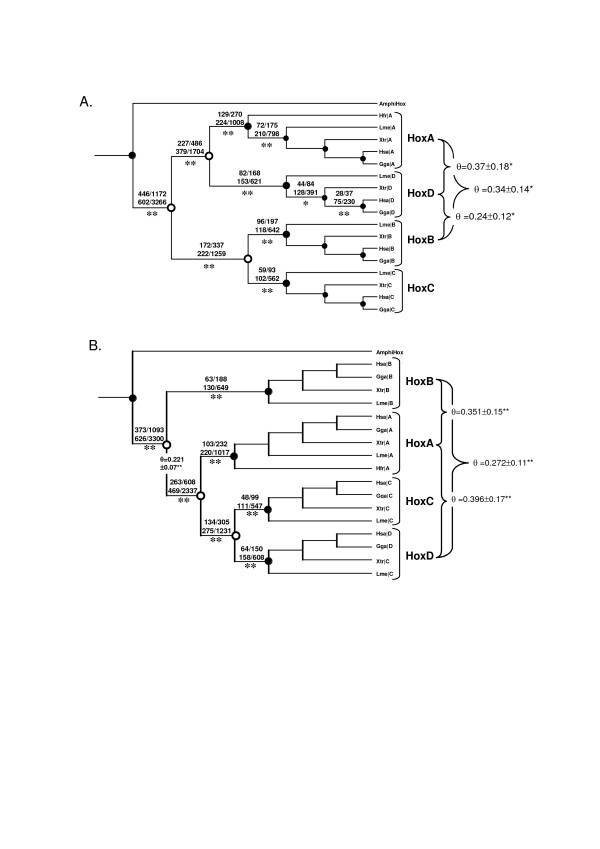
Relative Rate Ratio Tree and Coefficent of Functional Divergence. Numbers of replacement and silent, invariant and variant substitutions are shown above branches (RI/RV, SI/SV) for lineage with significant results indicating adaptive evolution. Coefficients of functional divergence (θ) estimated from DIVERGE are shown on the right; θ is shown on the internal branch separating HoxB from protoHoxACD for the divergence between HoxB and protoHoxACD. Results are shown for both the ((AD)(BC)) (A.) and (B(A(CD))) (B) topologies. *, p < 0.05; **, p < 0.001.

To test if 'cluster-specific' residues are functionally divergent we estimated the coefficient of functional divergence (θ), which measures the difference in evolutionary rate at amino acid sites between gene clusters. Rejection of the null hypothesis (θ = 0) is strong evidence for altered functional constraints after gene duplication (or speciation) [34]. We found significant evidence of type-I functional divergence for comparisons between HoxA, HoxB, and HoxD clusters (θ_I _= 0.24–0.37, p < 0.05; Figure 3) under the ((AD)(BC)) topology and under the (B(A(CD))) topology (θ_I _= 0.271–0.396, p < 0.05; Figure 3) at sites that differentiate paralog groups. We also found evidence of functional divergence of the HoxB cluster from the protoHoxACD cluster after the initial duplication event predicted under the (B(A(CD))) model (θ_I _= 0.221 ± 0.07, p < 0.05; Figure 3). Results were not significant for comparisons between HoxC and any other cluster (θ_I _= 0.001–0.029) under either duplication model. Comparisons between HoxB and HoxA to protoBC under the (B(A(CD))) model and protoAD to protoBC under the ((AD)(BC)) model, θ_I(B(A(CD))) _= 0.138–134 and θ_I((AD)(BC)) _= 0.001, respectively, were not significant. Type-I sites are defined as those with an amino acid that is conserved in one clade but variable in the sister clade, implying that the site is under structural/functional constraints in the first clade that is absent in the variable clade [34]. Type-I sites are located in the amino- and carboxy terminal ends of the homeodomain (outside of the 3 helices and in regions not predicted to be well structured) and in loop connecting helix 2 and 3.

Recently, a method has been developed to test for type-II functional divergence [35]. Type-II sites are those that are highly conserved in both clades but are fixed for amino acids with different biochemical properties between sister clades, implying these residues are responsible for functional differences between these groups. Although all paralog groups had at least one site with evidence of type-II divergence, all of which were radical amino acid substitutions (defined as a change in polarity or charge, but not size) of surface residues, the θ_II _values are extremely small (θ_II _= 0.001–0.062), highlighting the conservation between homeodomains from different clusters. These results are not unexpected given that this method calculates θ across all sites in an alignment and thus effectively averages site-wise θ values. With only ~4% of sites/cluster showing a pattern of type-II divergence in our concatenated alignment, it is not likely that the ~46 possible type-II sites have θ_II _values high enough to compensate for the extremely low θ_II _values of the over 900 sites with θ_II _effectively equal to zero.

### Accelerated evolution of homeodomains after cluster duplication

Several models of molecular evolution have been proposed to account for the preservation of duplicate genes. Although the details of each model can vary (see introduction), they differ in their predictions regarding the pattern of sequence evolution following gene duplication. The neo-functionalization and divergent selection models predict that the nonsynonymous substitution rate will be increased following gene duplication because of positive Darwinian selection in the gene acquiring the new function, while the Dykhuizen-Hartl and DDC models predict and increase in the substitution rate because of relaxed purifying selection. It is possible to distinguish between these models by comparing nonsynonymous (*d*_*N*_) to synonymous (*d*_*S*_) substitution rate (*d*_*N*_/*d*_*S *_= ω) with ω = 1, <1, and >1 indicating neutral evolution, purifying selection and directional selection.

Unlike the functional divergence methods developed by Gu [36,37], estimating selection using the *d*_*N*_/*d*_*S *_ratio is, by definition, dependent on the degree of divergence of the sequences under study. Thus, short sequences with a high degree of amino acid conservation but substantial synonymous site divergence may not contain enough signal to reliably obtain estimates of *d*_*N *_and *d*_*S*_. We assessed whether homeodomain sequences contained sufficient information for reliable rate estimates by examining the tree length statistic *S*, the number of nucleotide substitutions per codon. For individual paralog groups *S *range from 6.3–13.3 (average = 10.00), with tree length *d*_N _averaging 1.2 substitutions per nonsynonymous site and tree length *d*_S _averaging 18 substitutions per synonymous site along the tree. Interestingly, simulation studies [38] have shown that at levels of sequence divergence similar to our datasets, use of the χ^2 ^made the likelihood ratio test statistic (LRT) extremely conservative such that the type-I error rate is very small. Similarly, the power of the LRT to reject the null hypothesis even when it is false (type-II error) was found to be conservative even at medium to high levels of sequence divergence [38]. The power of the LRT increases as the number of sequences increases such that at 17 taxa power is nearly 100% [38], suggesting that our inclusion of at least 8–12 sequences (depending on the paralog group) helped alleviate loss of power from short conserved sequences. The simulation study results indicated that the optimal sequence divergence depends on the dataset and appears to be within the medium-to-high range [38]. Our data indicate that results based on estimates of *d*_*N *_and *d*_*S *_from this homeodomain dataset are reliable, if conservative.

To estimate the strength and kind of selection acting on Hox gene homeodomains, we used maximum likelihood methods to estimate the nonsysnonymous (*d*_*N*_) to sysnonymous (*d*_*S*_) substitution rate ratio [39,40]. The one ratio model is the simplest and provides a measure of the average strength and direction of selection acting on the gene throughout its history and can test if there was an increase in the rate of evolution after Hox cluster duplications. As expected, the *d*_*N*_/*d*_*S *_ratio for the homeodomains of all paralog groups is much less than 1 (0.0033–0.0359) highlighting the dominant role purifying selection plays on Hox gene evolution. To test if there was an increase in the nonsynonymous substitution rate following Hox cluster duplication we used a two ratios model that estimated separate ω's for post cluster duplication (ω_PCD_) and post speciation (ω_PS_) branches. Post cluster duplication branches evolved significantly faster (3–27x) than post speciation branches for 10 of 13 paralog groups; the remaining 3 paralog groups had ω_PCD _> ω_PS _but the results were not significant (Table 1). A more complex model that allowed each post duplication lineage to have separate *d*_*N*_/*d*_*S *_ratios from each other (the paralog 6 group for example: ω_PCD-A6_, ω_PCD-B6 _and ω_PCD-C6_) and post speciation (ω_PS_) branches was not better than the simple two-ratio model indicating that paralogs experienced similar selective forces after cluster duplication. These results are consistent with previous data from Hox5, Hox6 and Hox7 and indicate there was a period of rapid evolution of the homeodomain after Hox cluster duplication that could have been the result of either positive Darwinian selection or relaxed purifying selection.

**Table 1:** Likelihood paramater estimates under the lineage-specific models.

**Model**	**ℓ**	**PS-d_*N*_**	**PS-*d*_*S*_**	**PD-*d*_*N*_**	**PD-*d*_*S*_**	**ω_0_**	**ω_PCD_**	**Sig.**
Hox1								
One ratio	-707.88					0.0096		
**Two ratio**	**-705.14**					**0.0046**	**0.1250**	**P < 0.05**
Hox2								
One ratio	-800.02	0.0048	0.5967	0.0025	0.3130	0.0080		
**Two ratio**	**-798.08**	**0.0037**	**0.6313**	**0.0090**	**0.6700**	**0.0058**	**0.1334**	**0.05**
Hox3								
One ratio	-989.99	0.0042	1.1187	0.0003	0.0718	0.0037		
**Two ratio**	**-984.72**	**0.0034**	**1.3424**	**0.0050**	**0**	**0.0025**	A: (0/0) **B: (1.2/0)** **D: (1/0)**	**P << 0.01**
Hox4								
One ratio	-1573.85	0.0046	0.4348	0.0081	0.7736	0.0105		
**Two ratio**	**-1570.91**	**0.0034**	**0.4518**	**0.0151**	**0.5811**	**0.0076**	**0.0259**	**P < 0.05**
Hox5								
One ratio	-1015.97	0.0028	0.8522	0.0266	8.1561	0.0033		
Two ratio	-1014.86	0.0025	0.9172	0.0279	1.4231	0.0027	0.0196	n.s.
Hox6								
One ratio	-1309.19	0.0110	0.7089	0.0242	1.5604	0.0155		
**Two ratio**	**-1297.90**	**0.0096**	**0.7323**	**0.0337**	**0.5095**	**0.0131**	A: (0/0) **B: (3.5/0)** **C: (10/0)**	**P << 0.01**
Hox7								
One ratio	-701.50	0.0032	0.8238	0	0	0.0039		
**Two ratio**	**-696.90**	**0.0025**	**1.0950**	**0.0037**	**0**	**0.0023**	**(0.5/0)**	**P << 0.01**
Hox8								
One ratio	-1092.36	0.0055	0.3889	0.0129	0.9167	0.0141		
**Two ratio**	**-1089.88**	**0.0041**	**0.4206**	**0.0204**	**0.4981**	**0.0098**	**0.0409**	**P < 0.05**
Hox9								
One ratio	-1389.70	0.0042	0.8239	0.0112	2.1879	0.0101		
**Two ratio**	**-1377.10**	**0.0004**	**0.6179**	**0.0110**	**0.0950**	**0.0054**	**0.1156**	**P << 0.01**
Hox10								
One ratio	-1790.54	0.0118	0.3296	0.0204	0.5690	0.0359		
**Two ratio**	**-1783.60**	**0.0124**	**0.5521**	**0.0225**	**0**	**0.0224**	**(4.5/0)**	**P << 0.01**
Hox11								
One ratio	-1210.27	0.0054	0.5091	0.0132	1.2578	0.0105		
**Two ratio**	**-1206.53**	**0.0037**	**0.5339**	**0.0244**	**0.8452**	**0.0070**	**0.0288**	**P << 0.01**
Hox12								
One ratio	-1092.80	0.0144	0.6274	0.0545	2.3793	0.0229		
Two ratio	-1092.72	0.0141	0.6378	0.0563	1.8156	0.0222	0.031	n.s.
Hox13								
One ratio	-1745.34	0.0171	0.7425	0.0419	1.8193	0.0230		
**Two ratio**	**-1745.61**	**0.0160**	**0.7516**	**0.0496**	**0.9426**	**0.0212**	**0.0526**	**0.063**

Parameters that indicate rate accelerations are in bold. ℓ, likelihood of the model. Sig., significance of the model.

### Adaptive evolution of homeodomains after cluster duplication: Relative rate ratio tests

Although positive selection at the molecular level is most often tested using the *d*_*N*_/*d*_*S *_ratio, this method has several inherent limitations. The most problematic of which is when positive selection is acting at a limited number of sites while the majority are under strong purifying selection. Under these conditions *d*_*N *_will never become larger than *d*_*S *_and the signal for positive selection will be masked. In addition, when there is a large amount of sequence divergence between two nodes in a tree (site saturation) the accuracy of *d*_*S*_, and to a lesser extent *d*_*N*_, is greatly reduced. These two limitations of the *d*_*N*_/*d*_*S *_ratio to detect positive selection are particularly important for studying selective forces after Hox cluster duplications since very few sites (less than 15%) changed after duplication and the duplication events are relative ancient (about 560 MYA; ref), leading to substantial synonymous site divergence. Thus, even though we found evidence of accelerated rates of sequence evolution post cluster duplication, it is unlikely that the *d*_*N*_/*d*_*S *_ratio tests used above would be able to detect positive selection (ω > 1).

One complementary method that has been developed to compensate for some of limitations of the *d*_*N*_/*d*_*S *_ratio is the relative rate ratio test of Creevey and McInerney [41], which is an extension of the contingency test of neutrality proposed by Templeton [42] and McDonald and Kreitman [43]. Briefly, this method reconstructs ancestral sequences for each node in a phylogenetic tree using parsimony and identifies all substitutions that result in nonsynonymous and synonymous changes for each node. Substitutions are classified as replacement invariable (RI, i.e. nonsynonymous substitutions that are not substituted again in descendent lineages), replacement variable (RV, i.e. nonsynonymous substitutions that are substituted again in descendent lineages), silent invariable (SI, i.e. synonymous substitutions that are not substituted again in descendent lineages) and silent variable (RV, i.e. synonymous substitutions that are substituted again in descendent lineages).

Under neutral evolution the ratio of RI/RV will not be significantly different from SI/SV. Similarly, a period of relaxed purifying selection may increase RI/RV relative to SI/SV, but RI/RV will never be significantly greater than the neutral expectation given by SI/SV since the rate of replacement substitution can only exceed the rate of silent (neutral) substitution under positive selection. During an episode of positive selection, advantageous substitutions will become fixed in a lineage and remain invariant in descendent lineages, elevating the ratio of RI/RV relative to the neutral expectation given by SI/SV. Thus, when lineages are identified with a significantly greater RI/RV than SI/SV positive selection is indicated.

Using the relative rate ratio test to examine selective forces after cluster duplications identified that post-duplication lineages under the ((AD)(BC)) and the (B(A(CD))) models had significantly larger RI/RV than SI/SV (Figure 3 and Tables 2 and 3), indicating these duplication events were followed by adaptive evolution and supporting the results obtained with the *d*_*N*_/*d*_*S *_ratio tests and further suggesting that the increase in rates identified from the *d*_*N*_/*d*_*S *_ratio were due to positive selection.

**Table 2:** Results of the Creevey-McInerney test unde the ((AD)(BC)) topology.

**Branch**	**RI**	**RV**	**SI**	**SV**	**G-Value**	**G-test**	**Sig.**
branch 0	17	57	134	370	G = 0.443499	Gtest:0.900000	P > 0.500000
branch 1	31	105	187	583	G = 0.140809	Gtest:0.900000	P > 0.500000
**branch 2**	**72**	**175**	**210**	**798**	**G = 7.496351**	**Gtest:0.005000**	**P > 0.000000**
**branch 3**	**129**	**270**	**224**	**1008**	**G = 33.356056**	**Gtest:0.005000**	**P > 0.000000**
**branch 4**	**28**	**37**	**75**	**230**	**G = 8.448683**	**Gtest:0.005000**	**P > 0.000000**
**branch 5**	**44**	**84**	**128**	**391**	**G = 4.740880**	**Gtest:0.050000**	**P > 0.025000**
**branch 6**	**82**	**168**	**153**	**621**	**G = 17.139877**	**Gtest:0.005000**	**P > 0.000000**
**branch 7**	**227**	**486**	**379**	**1704**	**G = 54.900509**	**Gtest:0.005000**	**P > 0.000000**
branch 8	9	86	57	334	G = 1.802721	Gtest:0.200000	P > 0.100000
branch 9	35	137	102	470	G = 0.542717	Gtest:0.500000	P > 0.200000
**branch 10**	**96**	**197**	**118**	**642**	**G = 36.213978**	**Gtest:0.005000**	**P > 0.000000**
branch 11	2	44	57	289	G = 5.863089	Gtest:0.025000	P > 0.010000
branch 12	12	57	85	412	G = 0.003849	Gtest:0.990000	P > 0.950000
**branch 13**	**59**	**93**	**102**	**562**	**G = 37.628811**	**Gtest:0.005000**	**P > 0.000000**
**branch 14**	**172**	**337**	**222**	**1259**	**G = 77.613304**	**Gtest:0.005000**	**P > 0.000000**
**branch 15**	**446**	**117**	**602**	**3266**	**G = 101.06102**	**Gtest:0.005000**	**P > 0.000000**

RI, replacement invariant. RV, replacement variant. SI, synonymous invariant. SV, synonymous variant. The G-value and results of the G-test are shown along with the significant of the results.

**Table 3:** Results of the Creevey-McInerney test unde the ((AD)(BC)) topology.

**Branch**	**RI**	**RV**	**SI**	**SV**	**G-Value**	**G-test**	**Sig.**
branch 0	10	83	60	333	G = 1.304401	Gtest:0.500000	pvalue > 0.200000
branch 1	24	136	110	465	G = 1.477014	Gtest:0.500000	pvalue > 0.200000
**branch 2**	**63**	**188**	**130**	**649**	**G = 8.360222**	**Gtest:0.005000**	**pvalue > 0.000000**
branch 3	20	57	132	369	G = 0.005322	Gtest:0.950000	pvalue > 0.900000
branch 4	39	110	183	586	G = 0.376969	Gtest:0.900000	pvalue > 0.500000
branch 5	57	172	206	797	G = 2.035316	Gtest:0.200000	pvalue > 0.100000
**branch 6**	**103**	**232**	**220**	**1017**	**G = 25.201559**	**Gtest:0.005000**	**pvalue > 0.000000**
branch 7	9	42	60	286	G = 0.003351	Gtest:0.990000	pvalue > 0.950000
branch 8	12	71	94	399	G = 1.045448	Gtest:0.500000	pvalue > 0.200000
**branch 9**	**48**	**99**	**111**	**547**	**G = 17.036821**	**Gtest:0.005000**	**pvalue > 0.000000**
**branch 10**	**29**	**37**	**79**	**229**	**G = 8.251780**	**Gtest:0.005000**	**pvalue > 0.000000**
branch 11	43	87	139	386	G = 2.192354	Gtest:0.200000	pvalue > 0.100000
**branch 12**	**64**	**150**	**158**	**608**	**G = 7.819194**	**Gtest:0.005000**	**pvalue > 0.000000**
**branch 13**	**134**	**305**	**275**	**1231**	**G = 28.853481**	**Gtest:0.00500**	**pvalue > 0.000000**
**branch 14**	**263**	**608**	**495**	**2337**	**G = 61.973667**	**Gtest:0.00500**	**pvalue > 0.000000**
**branch 15**	**373**	**1093**	**626**	**3300**	**G = 60.691624**	**Gtest:0.00500**	**pvalue > 0.000000**

RI, replacement invariant. RV, replacement variant. SI, synonymous invariant. SV, synonymous variant. The G-value and results of the G-test are shown along with the significant of the results.

### Adaptive evolution of homeodomains after cluster duplication: d_N_/d_S_

The lineage-specific *d*_*N*_/*d*_*S *_model utilized above has been extended to account for variable *d*_*N*_/*d*_*S *_between sites and can detect positive selection at specific sites in specific lineages under appropriate conditions [44,45]. These branch-site models are ideal for detecting short episodes of positive selection that acted on a few sites while the majority of sites in the protein remained under purifying selection, as is likely to have occurred in the homeodomain after Hox cluster duplication. Applying branch-site models and to post cluster duplication (ω_PCD_) branches identified sites under positive selection after cluster duplication (Figure 3) in paralog groups 1–6, 9 and 13 (Table 4). Positive Sites were identified with posterior probabilities (PP) greater than 0.90 using the both the liberal Neive Empircal Bayes (NEB) and the more conservative Bayes Empirical Bayes (BEB) methods implemented in PAML3.15, although only the result of the BEB method is shown. In addition, two genes in the Hox3, 5, 6, and 13 paralog groups have evidence of positive selection, but the results are not statistically significant. The sites identified under positive selection are the same as those that show evidence of type-II functional divergence and map onto the molecular surface of the homeodomain, facing away from the DNA and in an orientation that would facilitate protein-protein interactions (Figure 2).

**Table 4:** Likelihood paramater estimates under the branch-site models.

**Model**	**ℓ**	**Parameters**	**Positive Sites**	**Sig.**
**Hox1**				
M1a	-707.37	p_0 _= 0.982, p_1 _= 0.018; ω_0 _= 0.0017, ω_1 _= 1	Not Allowed	
**MA**	**-697.04**	**P_0+1 _= 0.935, p_2 _= 0.065; ω_0/1 _= 0.0034/1, ω_2 _= 999**	**3 (PP > 0.99)**	**P << 0.001**
**Hox2**				
M1a	-800.91	p_0 _= 0.985, p_1 _= 0.015; ω_0 _= 0.0059, ω_1 _= 1	Not Allowed	
**MA**	**-794.72**	**p_0+1 _= 0.981, p_2 _= 0.019; ω_0/1 _= 0.0062/1, ω_2 _= 17.98**	**1 (PP > 0.95)**	**P < 0.005**
**Hox3**				
M1a	-1684.44	p_0 _= 0.979, p_1 _= 0.021; ω_0 _= 0.0223, ω_1 _= 1	Not Allowed	
**MA**	**-1647.20**	**p_0+1 _= 0.833, p_2 _= 0.167; ω_0/1 _= 0.0116/1, ω_2 _= 170.6**	**12 (PP > 0.95)**	**P << 0.001**
**Hox4**				
M1a	-1547.08	p_0 _= 0.987, p_1 _= 0.013; ω_0 _= 0.0057, ω_1 _= 1	Not Allowed	
MA	-1538.64	p_0+1 _= 0.987, p_2 _= 0.013; ω_0/1 _= 0.0046/1, **ω_2 _= 3.70**	**1 (PP > 0.99)**	P << 0.001
**Hox5**				
M1a	-1280.06	p_0 _= 1.0, p_1 _= 0.0; ω_0 _= 0.0119, ω_1 _= 1	Not Allowed	
**MA-A**	**-1272.42**	**p_0+1 _= 0.897, p_2 _= 0.103; ω_0/1 _= 0.008/1, ω_2 _= 1.11**	**1 (PP > 0.95)**	**P << 0.001**
**MA-B**	**-1273.35**	**p_0+1 _= 0.925, p_2 _= 0.075; ω_0/1 _= 0.0085/1, ω_2 _= 477.6**	**None Identified**	**P << 0.001**
**MA-C**	**-1269.82**	**p_0+1 _= 0.903, p_2 _= 0.097; ω_0/1 _= 0.0106/1, ω_2 _= 76.7**	**4 (PP > 0.90)**	**P << 0.001**
**Hox6**				
M1a	-1309.19	p_0 _= 1.0, p_1 _= 0.0; ω_0 _= 0.0155, ω_1 _= 1	Not Allowed	
MA	-1295.24	p_0+1 _= 0.754, p_2 _= 0.246; ω_0/1 _= 0.0108/1, **ω_2 _= 999**	**10 (PP > 0.90)**	P << 0.001
Hox7				
M1a	-698.86	p_0 _= 1.0, p_1 _= 0.0; ω_0 _= 0.0029, ω_1 _= 1	Not Allowed	
MA	-969.89	No Reliable Results		n.a.
Hox8				
M1a	-1889.89	p_0 _= 0.985, p_1 _= 0.015; ω_0 _= 0.011, ω_1 _= 1	Not Allowed	
MA	-1086.37	p_0+1 _= 0.949, p_2 _= 0.051; ω_0/1 _= 0.011/1, ω_*2 *_= *1.0*	*1 (PP > 0.95)*	P < 0.05
**Hox9**				
M1a	-1389.70	p_0 _= 1.0, p_1 _= 0.0; ω_0 _= 0.0101, ω_1 _= 1	Not Allowed	
MA-A	-1386.83	p_0+1 _= 0.878, p_2 _= 0.122; ω_0/1 _= 0.0089/1, ω_*2 *_= *1.0*	*None Identified*	P = 0.057
MA-B	-1380.29	No Reliable Results		n.a.
**MA-C**	**-1269.82**	**p_0+1 _= 0.903, p_2 _= 0.097; ω_0/1 _= 0.0106/1, ω_2 _= 76.7**	**4 (PP > 0.90)**	**P << 0.001**
MA-D	-1389.19	p_0+1 _= 0.9, p_2 _= 0.1; ω_0/1 _= 0.0095/1, ω_*2 *_= *1.0*	*1 (PP < 0.90)*	n.s.
Hox10				
M1a	-1747.75	p_0 _= 0.895, p_1 _= 0.105; ω_0 _= 0.0139, ω_1 _= 1	Not Allowed	
MA	-1747.20	p_0+1 _= 0.853, p_2 _= 0.47 ω_0/1 _= 0.0123/1, ω_*2 *_= *1.0*	*1 (PP < 0.90)*	n.s.
Hox11				
M1a	-1210.27	p_0 _= 0.982, p_1 _= 0.018; ω_0 _= 0.0017, ω_1 _= 1	Not Allowed	
MA	-1208.15	p_0+1 _= 0.973, p_2 _= 0.029; ω_0/1 _= 0.0092/1, ω_*2 *_= *1.0*	*None Identified*	n.s.
Hox12				
M1a	-1092.80	p_0 _= 1.0, p_1 _= 0.0; ω_0 _= 0.0229, ω_1 _= 1	Not Allowed	
MA	1092.52	p_0+1 _= 0.973, p_2 _= 0.027; ω_0/1 _= 0.0226/1, ω_*2 *_= *1.0*	*None Identified*	n.s.
**Hox13**				
M1a	-1747.34	p_0 _= 1.0, p_1 _= 0.0; ω_0 _= 0.023, ω_1 _= 1	Not Allowed	
**MA**	**-1731.97**	**p_0+1 _= 0.918, p_2 _= 0.082; ω_0/1 _= 0.022/1, ω_2 _= 6.96**	**3 (PP > 0.90)**	**P << 0.001**

Parameters that indicate positive selection are in bold. ℓ, likelihood of the model. Sig., significance of the model. Positive sites were identified using the Bayes empirical Bayes (BEB) method

While no sites under positive selection were identified in paralog groups 7, 8 and 10–12, a class of sites in each was identified with ω = 1 (Table 4). Given that the ability of likelihood models to detect sites with ω > 1 is an extremely difficult computational problem, it is possible that these sites actually experienced positive selection, but that the models are not able to identify ω > 1. An equally likely explanation that does not invoke positive selection is that the ω = 1 is an accurate estimate for the rate at this sites, and is actually indicative of relaxed functional constraints after duplication, that the sites have not been substituted again indicates they under strong purifying selection in post-speciation lineages, supporting a Dykhuizen-Hartl mechanism for their evolution.

### The structural basis of homeodomain evolution

**Figure 4:**
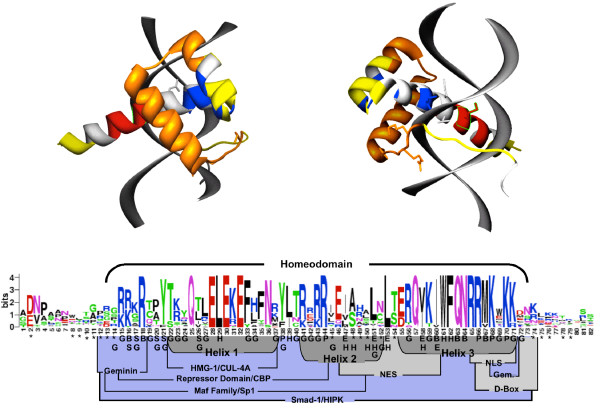
Function and Evolution of the Homeodomain. The structure of the homeodomain bound to DNA is shown as ribbon models. The location of the repressor domain is shown in orange, the nuclear localization signal in red, critical hydrophobic residues of the nuclear export signal in blue and positive sites in yellow. Only side chains of amino acids that make base contacts are shown. (C) Sequence logo of the Hox-gene homeodomain and surrounding amino acids. The overall height of the stacked amino acids indicates the sequence conservation at that position, while the height of symbols within the stack indicates the relative frequency of each amino acid at that position. The location of the homeodmain is shown above the logo. The location of protein-protein interaction regions are shown in blue (note that only some sites, not all sites, in blue actually participate in protein-protein interactions), sequence motifs are shown in light gray and the location of helices in dark gray. Sites identified under directional selection after cluster duplication are shown with an asterik (*). Sites with known functional information are shown: G, characteristic paralog-group residue; S, site that assists in binding site discrimination between paralog groups; B, site that makes base contacts; H, site that is part of the hydrophobic core; P, site that contacts the phosphate backbone; E, location of leucine and isoleucine residues critical for the nuclear export signal.

To gain a better understanding of how functional constraints on the homeodomain relate to sequence divergence, we generated a sequence logo [46,47] from the multiple sequence alignment of Hox-gene homeodomains and mapped the location of sites under positive selection and residues with known functions onto the logo and the crystal structure of the homeodomain bound to DNA (Figure 4). Adaptive/functionally divergent sites are grouped into three discrete regions of the homeodomain: the extreme amino and carboxy terminal arms just outside of the homeodomain proper and in the C-terminal end of helix-2 extending into the loop connecting helix-2 and helix-3.

The repressor domain, where the majority of protein interactions have been found, and helix-3 are free from positive sites, likely reflecting conserved functions shared by all Hox genes. Several proteins have been shown to bind in the repressor domain including the CREB binding protein (CBP) [48], high mobility group protein 1 (HMG1) [49], members of the Maf family of basic-leucine zipper (bZip) activators [50], and geminin [51]. This region also overlaps with the Sp1 transactivation region [52]. In addition to characterized protein-protein interactions, the repressor domain also contains the majority of 'characteristic-residues' that distinguish cognate groups from each other indicating the majority of sites in this region were already under functional constraints after the tandem duplications which created the Hox gene cluster and were not available to be targets of adaptive selection after cluster duplication. Interestingly, the first 3 sites of the geminin-binding region were under directional selection in different paralog groups, including radical amino acid substitutions, suggesting selection to modulate geminin binding between paralog group members.

Mapping functionally divergent amino acid sites and sites under positive selection in and around helix-2 onto the logo and crystal structure shows that purifying selection has acted to preserve hydrophobic/aliphatic residues critical for the nuclear exportation signal [53] and positive selection has acted exclusively on sites that occur at the molecular surface. These sites form a small cluster at the posterior end of helix-2 in a prime location for protein-protein interactions. Beyond the ultra-conserved helix-3, which also contains the nuclear localization signal [54], sites under positive selection have been identified in an unstructured connecting loop leading to additional structures in the carboxy terminus. The amino-terminal arm of the homeodomain, which confers functional specificity on Hox proteins, contains the majority of sites under positive selection suggesting that selection has acted to modify functional specificity between paralogs. This region appears to be unstructured and is a prime target for protein-protein interaction sites. This pattern of purifying and positive selection suggests that after Hox cluster duplications, selection acted on protein-protein interaction sites in such a way that ancestral functions were maintained while the acquisition of novel protein interaction partners driven was driven by selection on non-constrained amino acids. These derived interactions could be those responsible for novel Hox gene functions in vertebrates.

## Conclusion

The homeodomain serves multiple functions in addition to DNA-binding, including containing nuclear localization and export signals, transcriptional activation and repression domains and other protein-protein interaction sites [33,54]. These functions combine to impose severe limitations on the degree of sequence divergence that can be accommodated by the homeodomain of Hox genes. Even with these constraints, however, the relatively small set of amino acids that were free to diverge after cluster duplication were subject to positive selection. Although the Hox cluster duplications are relatively ancient (450 MYA), complicating the detection of positive selection, we find congruence between multiple methods a strong indicating that our results are reliable. These results support an important role for the action of positive Darwinian selection in the divergence of Hox genes after cluster duplications, particularly at sites that distinguish paralog groups ('cluster-specific' residues).

Nearly all 'cluster-specific' residues map onto the molecular surface of the homeodomain, similar to the paralog group specific sites [33], suggesting changes in amino acid properties could influence interaction of the homeodomain with other proteins. Cofactor associations are important for Hox proteins and most other transcription factor functions; these protein-protein interactions occur at the molecular surface through the formation of hydrophobic and ionic bonds and other intermolecular interactions such as salt bridges and van der Vaals forces. Thus, changes in the physicochemical properties of amino acids participating in these bonds could disrupt preexisting interactions and/or lead to new interactions. These changes could provide a selective advantage for maintaining duplicate genes through the origin of novel protein-protein interactions (effectively reducing degeneracy between paralogs) leading to new gene functions.

## Methods

The homeodomain of Hox genes was identified from BLAST searches of the nr database at NCBI. At least four members of each gene from diverse taxa were included in the dataset. The sequences were aligned based on the translated amino acid sequences with Se-Al v2.0, alignments were simple given the high degree of sequence conservation within paralog groups. Regions of ambiguous alignment just outside of the homeodomain but within exon 2 were excluded. Most alignments ranged from 70–82 amino acids. The alignment is available from V.J.L. and has been deposited in TREEBASE.

We used codon-based maximum likelihood models of coding sequence evolution implemented in CODEML in the PAML package of programs (version 3.15) to test for lineages and amino acid sites under positive selection. Sites were classified as being under positive selection if they were identified from the Bayes Empirical Bayes (BEB) method with a posterior probability of greater than 0.90. The branching order of the Hox cluster duplications is still debated (refs), but our analyses suggest that the most likely topologies are ((AD)(BC)) and (B(A(CD))) (a detailed analysis of Hox cluster duplication history is beyond the scope of this paper and will be presented elsewhere). We used 2 alternate trees to test for selection: ((AD)(BC)) and (B(A(CD))) and found no significant differences between the results of these different topologies. Functional divergence was tested with DIVERGE alpha1.2 (obtained from X. Gu). We also used the relative rate ratio test of Creevey and McInerny [41] implemented in the program CRANN to test for adaptive evolution. Both DIVERGE and CRANN analyses used the 2 alternate topologies discussed above.

## Authors' contributions

VJL designed and carried out the project, and wrote the manuscript with contributions from JJR and GPW. JJR provided information on protein-protein interaction sites and GPW provided biological insights and guidance during the course of this study.
